# Marked potentiation of cell swelling by cytokines in ammonia-sensitized cultured astrocytes

**DOI:** 10.1186/1742-2094-7-66

**Published:** 2010-10-13

**Authors:** Kakulavarapu V Rama Rao, Arumugam R Jayakumar, Xiaoying Tong, Veronica M Alvarez, Michael D Norenberg

**Affiliations:** 1 Department of Pathology, University of Miami Miller School of Medicine, Miami, FL 33125, USA; 2 Department of Biochemistry & Molecular Biology, University of Miami Miller School of Medicine, Miami, FL 33125, USA; 3 Veterans Affairs Medical Center, Miami, Fl, 33125, USA

## Abstract

**Background:**

Brain edema leading to high intracranial pressure is a lethal complication of acute liver failure (ALF), which is believed to be cytotoxic due to swelling of astrocytes. In addition to the traditional view that elevated levels of blood and brain ammonia are involved in the mechanism of brain edema in ALF, emerging evidence suggests that inflammatory cytokines also contribute to this process. We earlier reported that treatment of astrocyte cultures with a pathophysiological concentration of ammonia (5 mM NH_4_Cl) resulted in the activation of nuclear factor-kappaB (NF-κB) and that inhibition of such activation diminished astrocyte swelling, suggesting a key role of NF-κB in the mechanism of ammonia-induced astrocyte swelling. Since cytokines are also well-known to activate NF-κB, this study examined for additive/synergistic effects of ammonia and cytokines in the activation of NF-κB and their role in astrocyte swelling.

**Methods:**

Primary cultures of astrocytes were treated with ammonia and cytokines (TNF-α, IL-1, IL-6, IFN-γ, each at 10 ng/ml), individually or in combination, and cell volume was determined by the [^3^H]-*O*-methylglucose equilibration method. The effect of ammonia and cytokines on the activation of NF-κB was determined by immunoblots.

**Results:**

Cell swelling was increased by ammonia (43%) and by cytokines (37%) at 24 h. Simultaneous co-treatment with cytokines and ammonia showed no additional swelling. By contrast, cultures pretreated with ammonia for 24 h and then exposed to cytokines for an additional 24 h, showed a marked increase in astrocyte swelling (129%). Treatment of cultures with ammonia or cytokines alone also activated NF-κB (80-130%), while co-treatment had no additive effect. However, in cultures pre-treated with ammonia for 24 h, cytokines induced a marked activation of NF-κB (428%). BAY 11-7082, an inhibitor of NF-κB, completely blocked the astrocyte swelling in cultures pre-treated with ammonia and followed by the addition of a mixture of cytokines.

**Conclusion:**

Our results indicate that ammonia and a mixture of cytokines each cause astrocyte swelling but when these agents are added simultaneously, no additive effects were found. On the other hand, when cells were initially treated with ammonia and 24 h later given a mixture of cytokines, a marked potentiation in cell swelling and NF-κB activation occurred. These data suggest that the potentiation in cell swelling is a consequence of the initial activation of NF-κB by ammonia. These findings provide a likely mechanism for the exacerbation of brain edema in patients with ALF in the setting of sepsis/inflammation.

## Background

Hepatic encephalopathy (HE) is a neurological disorder that presents in chronic and acute forms. Chronic HE is a neuropsychiatric disorder which commonly occurs in the setting of alcoholic cirrhosis and is associated with changes in personality, altered mood, decline in the intellectual capacity and abnormal muscle tone [1]. Acute HE (acute liver failure, ALF) usually occurs following viral-mediated hepatitis, acetaminophen toxicity, and exposure to other hepatotoxins. ALF often presents with the abrupt onset of delirium, seizures and coma and has a high mortality rate (80-90%) [2]. A major component of ALF is the development of brain edema leading to increased intracranial pressure and brain herniation, ultimately resulting in death [3]. There is currently no satisfactory treatment for the edema in ALF except for an emergency liver transplantation [4].

The preponderance of evidence indicates that the brain edema in ALF is "cytotoxic", i.e., due to astrocyte swelling [5-9]. It has traditionally been considered that ammonia represents a major etiological factor in the development of neurological abnormalities associated with severe liver failure, including cytotoxic brain edema/astrocyte swelling [6,10]. Astrocytes primarily detoxify ammonia by converting glutamate into glutamine through a reaction mediated by glutamine synthetase, an enzyme predominantly present in astrocytes [11]. Accordingly, several studies document that a pathophysiological concentration of ammonia results in astrocyte swelling in culture [12-14], brain slices [15,16] and *in vivo *models of hyperammonemia [6,17,18]. The mechanism by which ammonia and the subsequent production of glutamine leads to astrocyte swelling/brain edema is incompletely understood, but appears to involve the development of oxidative/nitrosative stress, the mitochondrial permeability transition and activation of mitogen activated protein kinases. For review, see [19].

In addition to ammonia, emerging evidence suggests that proinflammatory cytokines, likely derived from liver necrosis and/or sepsis (the latter a common complication in ALF) play an important role in brain edema formation in ALF. In support of this view, blood levels of TNF-α, IL-1β and IL-6 were found elevated in patients with ALF who had concurrent infections [20-22]. Additionally, induction of endotoxemia was shown to exacerbate brain edema in an experimental model of hyperammonemia [23].

While the above studies strongly suggest the involvement of inflammatory cytokines in the brain edema of ALF, the effect of various cytokines on astrocyte swelling has thus far not been investigated. The present study therefore examined the effect of various inflammatory cytokines on astrocyte swelling, as well as investigated the potential additive/synergistic interactions between ammonia and cytokines on cell swelling in cultured astrocytes.

We recently documented that exposure of cultured astrocytes to a pathophysiological concentration of ammonia results in the activation (nuclear translocation) of the transcriptional factor NF-κB and that inhibition of such activation caused a reduction in ammonia-induced astrocyte swelling [24]. As cytokines are also well-known to activate NF-κB, we examined whether ammonia and cytokines exert additive/synergistic effects in the activation of NF-κB and whether such activation contributes to potential additive/synergistic effects on astrocyte swelling. Our studies show that a mixture of cytokines cause cell swelling in cultured astrocytes, and that sensitization of cultures with ammonia prior to treatment with cytokines caused a marked potentiation of both the activation of NF-κB and astrocyte swelling.

## Methods

### Materials

3-*O*-methyl glucose was purchased from American Radio chemicals Inc, St. Louis, MO. Recombinant rat TNF-α, IL-β, IL-6 and IFN-γ, and all other reagents were obtained from Sigma-Aldrich, St. Louis, MO.

### Astrocyte cultures

Primary cultures of astrocytes were prepared from cerebral cortices of 1-2 day old rats as described earlier [25]. Briefly, cortices were freed of meninges, minced and dissociated by trituration, passed through sterile nylon sieves and then placed in Dulbecco's modified Eagle medium (DMEM) containing penicillin, streptomycin, and 15% fetal bovine serum. Approximately 0.5 × 10^6 ^cells were seeded in 35 mm culture plates and maintained at 37°C in an incubator equilibrated with 5% CO_2 _and 95% air. Cultures consisted of 95-99% astrocytes based on immunohistochemical staining with glial fibrillary acidic protein. After 14 days, cultures were treated and maintained with dibutyryl cAMP to enhance cell differentiation [26]. Three to four-week-old cells were used in these experiments.

### Cytokine and ammonia treatment

Astrocyte cultures were treated with rat recombinant TNF-α, IL-1β, IL-6 and IFN-γ, all 10 ng/ml each, either individually or in combination for 3-24 h. For ammonia treatment studies, cultures were exposed to a pathophysiological concentration of ammonia (5 mM NH_4_Cl); such concentration of ammonia is found in brains of experimental animals with ALF [27-29]. To examine the effect of prior treatment with ammonia followed by exposure to cytokines, cultures were first treated with ammonia for 24 h, at which time a mixture of cytokines was added for an additional 24 h.

### Cell volume determination

Cell volume (intracellular water space) was determined using the 3-*O*-methyl-[^3^H]-glucose (OMG) method as described by Kletzien et al. [30] and modified for astrocyte cultures by Norenberg et al. [12]. In brief, cultured astrocytes were incubated with [^3^H] OMG (1 mM containing 1 μCi of radioactive OMG) (Sigma Aldrich, St. Louis, MO), and at the end of incubation, a small aliquot of medium was saved for specific activity determination. Cultures were washed three times with ice-cold buffer containing 290 mM sucrose, 1 mM Tris-nitrate (pH 7.4), 0.5 mM calcium nitrate and 0.1 mM phloretin. Cells were harvested in 0.5 ml of 1 N sodium hydroxide. Radioactivity was converted to intracellular water space and expressed as μl/mg cell protein. Protein content was determined by the BCA method (Pierce, Rockford, IL).

### Preparation of nuclear extract and Western blots for NF-κB

The nuclear extract was prepared as described previously [24]. In brief, astrocytes were harvested in 1 mL PBS and centrifuged at 735 *g *for 3 min at 4°C. The cell pellet was suspended in a buffer containing 10 mM HEPES (pH 7.9), 10 mM KCl, 0.1 mM EDTA, 0.1 mM EGTA, 1 μM dithiothreitol and a complete protease inhibitor cocktail (Roche, Mannheim, Germany); incubated on ice for 15 min; 15 μL of 10% NP-40 (Roche Diagnostics Corp., Indianapolis, IN) was added and the sample was vortexed thoroughly for 40 s and centrifuged at 735 *g *for 3 min at 4°C. The resulting nuclear pellet was resuspended in a buffer containing 20 mM HEPES (pH 7.9), 0.4 M NaCl, 1 mM EDTA, 1 mM EGTA, 1 μM dithiothreitol and protease inhibitors and centrifuged at 16, 000 *g *for 5 min at 4°C. The supernatant was loaded on SDS polyacrylamide gels and Western blots were performed as described previously [24].

Antibodies against NF-κB-p65 (H-286) (Santa Cruz Biotechnology Inc, Santa Cruz, CA) and the nuclear marker lamin (Cell Signaling Technology, Beverly, MA) were used at 1:750 and 1:1000 dilutions, respectively. Horseradish peroxidase-conjugated anti-rabbit secondary antibody (1:1000) (Vector Laboratories, Burlingame, CA) was used. Membranes were visualized using an enhanced chemiluminescence reagent. The optical density of the bands was measured with the Chemi-Imager digital imaging system (Alpha Innotech Corp, San Leandro, CA) and the results were quantified with the Multi-Analyst software (Sigma Scan Pro 5; SPSS Inc., Chicago, IL) as a proportion of the signal of house-keeping protein band (lamin).

### Statistical analysis

The data were expressed as means ± SEM and analyzed by ANOVA followed by Neuman-Keuls multiple-range test. A p < 0.05 was considered statistically significant.

## Results

### Effect of cytokines on astrocyte cell volume

**Figure 1:**
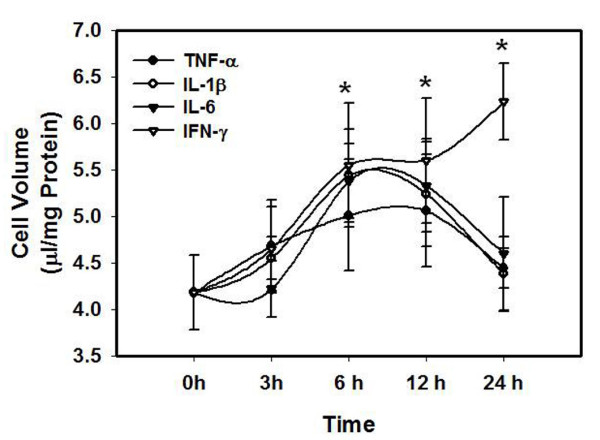
**Time course of astrocyte cell volume following treatment with individual cytokines (TNF-α, IL-1β, IL-6 and IFN-γ)**. Cultures were treated with 10 ng/ml concentration of each cytokine. * vs. Control, p < 0.05. Values in each group are mean ± S.E.M of 5 individual culture plates taken from 3 separate seedings (n = 15).

**Figure 2:**
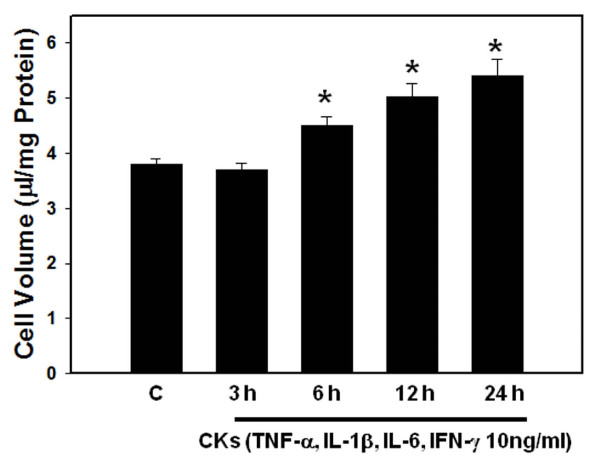
**Time course of astrocyte cell volume following treatment with a mixture of cytokines (TNF-α, IL-1β, IL-6 and IFN-γ, 10 ng/ml each)**. * vs. Control, p < 0.05. CKs, cytokines. Values in each group are mean ± S.E.M of 5 individual culture plates taken from 3 separate seedings (n = 15).

Treatment of cultures with individual cytokines TNF-α, IL-1β, IL-6 and IFN-γ (10 ng/ml each) showed a time-dependent increase in astrocyte swelling: at 3 h no astrocyte swelling was observed, while at 6 h, a 27-32% cell swelling was identified after treatment with all cytokines, which persisted for up to 12 h (Figure 1). At 24 h cell swelling was non-significant (14%) in cultures treated with TNF-α, IL-1β, IL-6. However, cultures treated with IFN-γ continued to display cell swelling for up to 24 h (44 ± 3%). We next examined the effect of a combination of cytokines (TNF-α, IL-1β, IL-6 and IFN-γ; 10 ng/ml; 24 h) and found that such treatment resulted in a 38 ± 2% increase in astrocyte cell volume (Figure 2); i.e., there was no difference in the degree of astrocyte swelling, when cultures were treated with cytokines individually or as a mixture.

### Effect of cytokines plus ammonia on astrocyte cell volume

**Figure 3:**
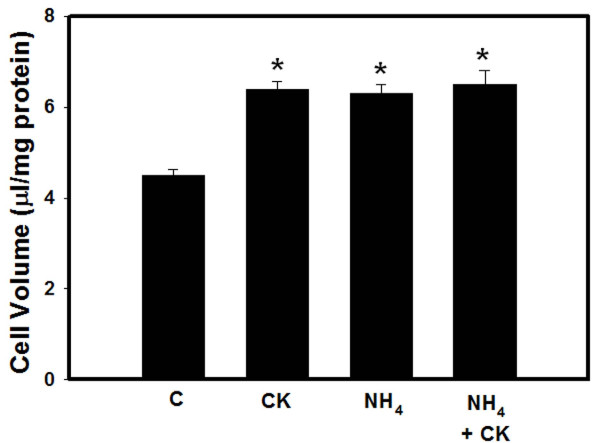
**Effect of a co-treatment of ammonia and a mixture of cytokines on cell volume (24 h) in cultured astrocytes**. * vs. Control, p < 0.05. Values in each group are mean ± S.E.M of 4 individual culture plates taken from 3 separate seedings (n = 12).

Ammonia is known to cause astrocyte swelling [12]. We therefore examined whether any additive/synergistic effects exist between ammonia and cytokines in the induction of cell swelling. Simultaneous co-treatment (24 h) of cultures with ammonia and cytokines showed no additive effects on astrocyte swelling (Figure 3).

**Figure 4:**
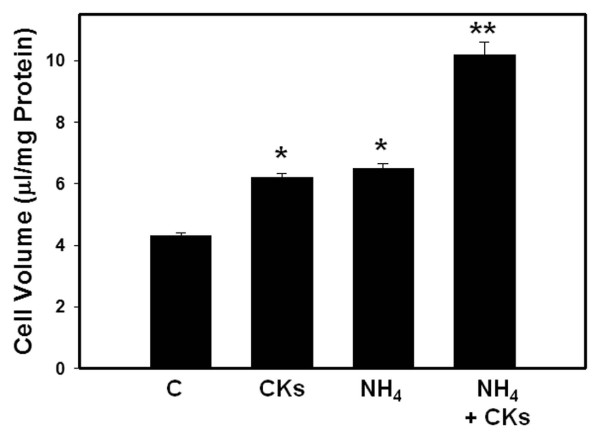
**Synergistic effects on astrocyte cell volume by a pre-treatment of cultures (sensitization) with ammonia followed by treatment with a mixture of cytokines on astrocyte cell volume (24 h)**. * vs. Control, p < 0.05; ** vs. CKs and NH_4_, p < 0.05. Values in each group are mean ± S.E.M of 5 individual culture plates taken from 3 separate seedings (n = 15).

Since in the clinical setting of ALF, elevated ammonia levels in blood/brain typically precedes inflammatory responses that might arise from liver necrosis and/or sepsis, we examined whether exposure of cytokines to ammonia-sensitized astrocytes showed additive or synergistic effects on astrocyte swelling. Cultures were treated with ammonia for 24 h and then exposed to a mixture of cytokines for an additional 24 h. Such treatment resulted in a marked potentiation of astrocyte swelling (129 ± 6%) (Figure 4) as compared to a simultaneous treatment of cultures with ammonia and a mixture of cytokines (Figure 3).

### Effect of a mixture of cytokines on the activity of NF-κB

**Figure 5:**
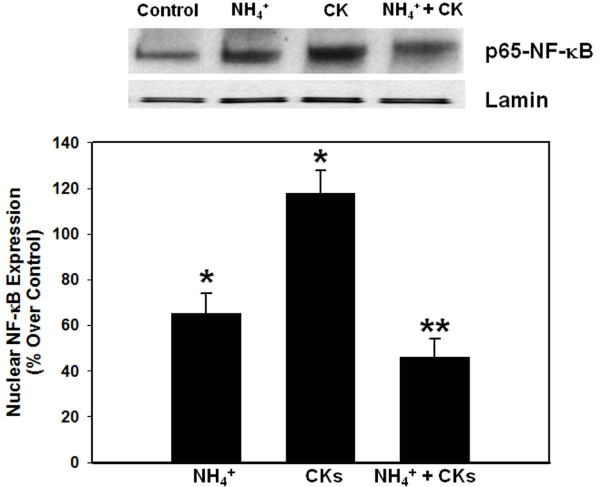
**Effect of a co-treatment of ammonia and a mixture of cytokines on the activation (nuclear translocation) of NF-κB in cultured astrocytes**. * vs. Control, p < 0.05; ** vs. CKs and NH_4_, p < 0.01. Values in each group are mean ± S.E.M of 3 individual culture plates taken from 3 separate seedings (n = 9).

**Figure 6:**
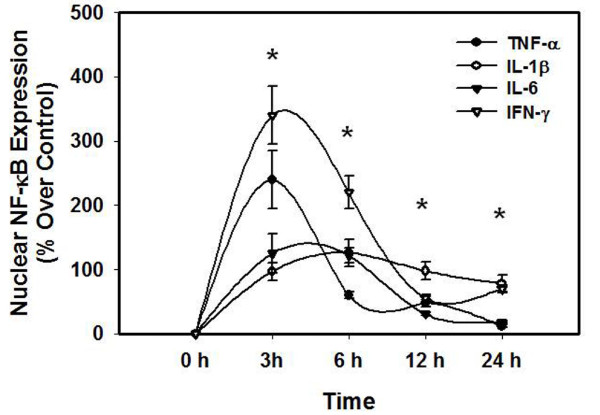
**Time course of NF-κB activation following treatment with individual cytokines (TNF-α, IL-1β, IL-6 and IFN-γ, 10 ng/ml each)**. * vs. Control, p < 0.05. Values in each group are mean ± S.E.M of 3 individual culture plates taken from 3 separate seedings (n = 9).

We recently documented that treatment of cultured astrocytes with ammonia results in the activation of the transcriptional factor NF-κB as shown by an increase in its nuclear translocation, and that inhibition of such activation significantly attenuated ammonia-induced astrocyte swelling [24], indicating an important role of NF-κB in such swelling. Since cytokines are also known to activate NF-κB [31], we examined the effect of a mixture of cytokines on NF-κB activation. Treatment of cultures with ammonia resulted in a 100 ± 10% (p < 0.01) increase in the activation of NF-κB. Likewise, a mixture of cytokines significantly increased (96 ± 8%) the activation of NF-κB (Figure 5). Treatment of cultures with individual cytokines also resulted in NF-κB activation in a time-dependent manner but the degree of such activation varied with each cytokine (Figure 6). However, a good correlation was observed between astrocyte swelling and the activation of NF-κB by individual cytokines (Figures 1 and 6).

### Effect of a combination of ammonia and a mixture of cytokines on the activity of NF-κB

**Figure 7:**
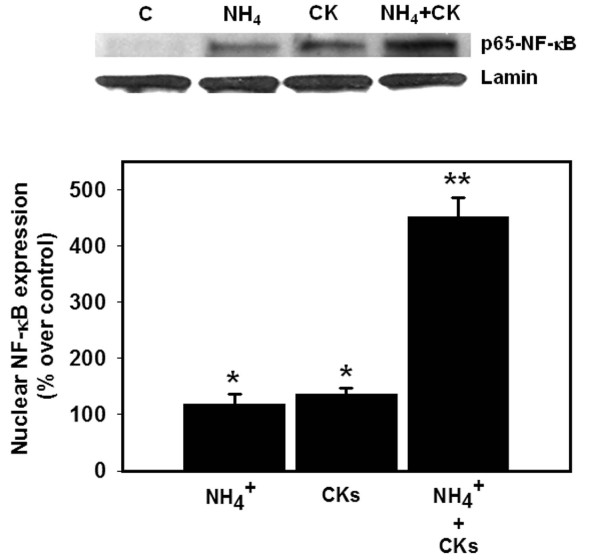
**Synergistic effects on NF-κB activation by a 24 h treatment of cultures with ammonia followed by treatment with mixture of cytokines on astrocyte cell volume (24 h)**. * vs. Control, p < 0.05; ** vs. CKs and NH_4_, p < 0.01. Values in each group are mean ± S.E.M of 3 individual culture plates taken from 3 separate seedings (n = 9).

Similar to the findings of astrocyte swelling, a simultaneous co-treatment (24 h) of cultures with ammonia and cytokines showed no additive/synergistic effects on NF-κB activation (Figure 5). However, in cultures previously treated with ammonia (24 h), the subsequent exposure of cells to a mixture of cytokines for an additional 24 h resulted in a marked increase in the activation of NF-κB (428%) (Figure 7). These findings strongly suggest that the marked potentiation of astrocyte swelling by a pre-treatment with ammonia followed by a treatment with a mixture cytokines is a consequence of NF-κB activation.

### Effect of BAY 11-7082 on the astrocyte swelling produced by ammonia and cytokines

**Figure 8:**
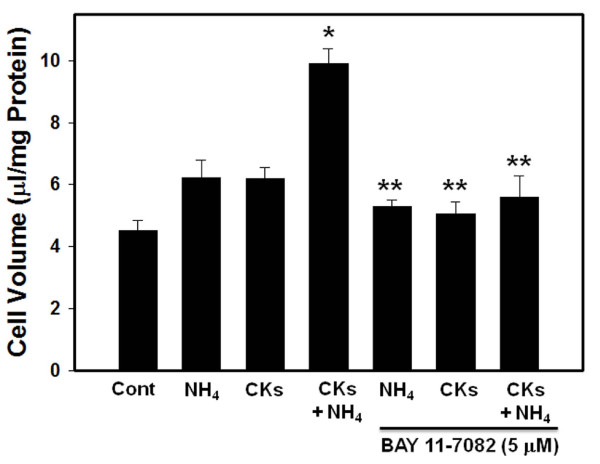
**Effect of inhibition of NF-κB activation by BAY 11-7082 on astrocyte cell volume (24 h) induced by a mixture of cytokines and ammonia**. * vs. Control, p < 0.05; ** vs. CKs; NH_4 _and NH_4 _+ CKs, p < 0.05. Values in each group are mean ± S.E.M of 5 individual culture plates taken from 3 separate seedings (n = 15).

We earlier documented that BAY 11-7082, an inhibitor of NF-κB, significantly diminished ammonia-induced astrocyte swelling [24]. The present study found that pretreatment of cultures with BAY 11-7082 diminished ammonia-induced astrocyte swelling (55%), which is consistent with our previous observations [24]. Additionally, BAY 11-7082 completely blocked the astrocyte swelling caused by a mixture of cytokines, as well as completely blocked the potentiation in cell swelling in cultures exposed to ammonia followed 24 later by the addition of a mixture of cytokines (Figure 8).

## Discussion

This study demonstrates that a combination of inflammatory cytokines, including TNF-α, IL-1β, IL-6 and IFN-γ, as well as cytokines given individually, can induce astrocyte swelling. Simultaneous co-treatment of astrocytes with a pathophysiological concentration of ammonia and cytokines failed to elicit any additive/synergistic effect. However, treatment (24 h) of astrocyte cultures with ammonia prior to exposure to a mixture of cytokines for an additional 24 h resulted in a marked potentiation of astrocyte swelling. Treatment of cultures with a combination of cytokines, or cytokines given individually, resulted in the activation of NF-κB. Additionally, we observed that sensitization of cultures with ammonia before exposure to a mixture of cytokines resulted in a marked increase in the activation of NF-κB, similar to that observed with cell swelling. Treatment of cultures with BAY 11-7082, an inhibitor of NF-κB, completely blocked the potentiation effects of cell swelling of astrocytes sensitized by ammonia and subsequently treated with cytokines. Altogether, these data indicate that ammonia and cytokines independently cause the activation of NF-κB and astrocyte swelling, and that pretreatment of cultures with ammonia sensitizes these cells to the effect of cytokines, resulting in a marked increase in both NF-κB activation and cell swelling, indicating a critical role of NF-κB in the potentiation of astrocyte swelling.

While ammonia continues to play a prominent role in the mechanism of brain edema in ALF, the findings of this study on the role of cytokines in the mechanisms of astrocyte swelling are highly pertinent and add to a growing body of evidence suggesting that inflammation and inflammatory cytokines also contribute to the brain edema in ALF [32-36]. The generation of cytokines is a consequence of extensive liver necrosis as well as infection/sepsis which frequently complicates ALF [20,37]. Additionally, lipopolysaccharide, a well-known inducer of inflammation [38], was shown to exacerbate brain edema in an experimental model of hyperammonemia [23]. Our observations on the marked potentiation of cell swelling by priming of cells with ammonia followed by treatment with cytokines in cultured astrocytes correspond well with the above clinical findings.

The present study also showed that TNF-α, IL-1β, or IL-6 given individually induces astrocyte swelling. Following treatment with these cytokines, astrocyte swelling was observed at 6 h and persisted for up to 12 h; however, by 24 h no swelling was detected. These findings suggest that cell swelling produced by cytokines is an early but transient event. Consistent with our results, one study documented that treatment of cultured astrocytes with TNF-α for 48 h did not affect astrocyte cell volume [39]. Our findings are also consistent with a recent report showing that in transgenic mice deficient in TNF-α, IL-1β and IL-6 receptors, ALF results in a lesser degree of brain edema as compared to levels achieved in wild-type mice [40]. To the best of our knowledge, this study documents for the first time that various inflammatory cytokines cause cell swelling in cultured astrocytes.

In contrast to TNF-α, IL-1β and IL-6, IFN-γ caused cell swelling which persisted for up to 24 h. The reason for the different pattern of swelling observed with IFN-γ is not known. The effect of IFN-γ, however, is at variance with a recent report noting that mice deficient in IFN-γ receptors do not show a reduction in brain edema following the induction of ALF [40]. While the reason for the differences regarding the role of IFN-γ in cell swelling/brain edema is not known, studies by Bemeur et al [40] noted that brain levels of IFN-γ were not elevated in wild-type mice after the administration of azoxymethane, a model of ALF that results in death within 16-18 h. By contrast, using a rat model of ALF induced by the hepatotoxin thioacetamide, which displays a more protracted clinical course (death occurring at 72-80 h), we found a modest increase (32%) in IFN-γ in brain, while the levels of TNF-α, IL-1β were elevated by 100-140% (unpublished observations). It is possible that differences in models of ALF, particularly in the rate of clinical progression and the degree of severity of liver failure, may be critical in the differential effects relative to the role of IFN-γ in the brain edema associated with ALF.

The present study additionally showed that a mixture of all four cytokines resulted in astrocyte swelling; however, the degree of swelling caused by a combination of cytokines did not differ from that exerted by individual cytokines. The reason for the absence of a potential additive effect on cell swelling when cytokines were added as a mixture is not clear. It is possible that negative interactions among cytokines may have precluded an additive effect. Consistent with this view, it has been shown that treatment of cultured astrocytes with TNF-α and IFN-γ showed no additive effects on the expression of complement (C3) genes, although individually these cytokines significantly enhanced C3 gene expression [41]. Additionally, IFN- γ has been shown to antagonize the effect of TNF-α on the production of chemokines in human astrocytes [42].

A notable finding of this study was that treatment of cultures with ammonia for 24 h prior to treatment with a mixture of cytokines for additional 24 h resulted in a marked potentiation of astrocyte swelling. By contrast, co-treatment with ammonia and cytokines did not result in such potentiation on astrocyte swelling. These data suggest that ammonia sensitizes astrocytes to the effect of cytokines, thereby resulting in a potentiation of cell swelling. This pattern parallels the clinical findings in ALF whereby the initial hyperammonemia is often followed by an inflammatory response as a consequence of liver necrosis and/or sepsis [32,33].

The mechanism by which prior sensitization of cultures with ammonia followed by treatment with a mixture of cytokines results in a marked increase in astrocyte swelling is not well understood. It is, however, reasonable to suggest that sensitization of cultures with ammonia facilitates a process by which cytokines potentiate their effects on astrocyte swelling. Pertinent to this view, it was recently demonstrated that exposure of cultured astrocytes to ammonia results in the activation of NF-κB as early as 12 h. Such activation appears to be mediated by oxidative/nitrosative stress (ONS), and activation of mitogen activated protein kinases (MAPKs) [24]. It is therefore possible that the initial activation of NF-κB by ammonia (24 h ammonia sensitization) promotes a synergistic effect on NF-κB activation after the addition of cytokines. This sequence is plausible as cytokines are well-known to activate NF-κB [31,43-45].

A parallel pattern observed between cell swelling and NF-κB activation supports the proposed key role of NF-κB in such astrocyte swelling. Thus, the time-course of activation of NF-κB by TNF-α, IL-1β and IL-6, IFN-γ, correlate with the time-course of astrocyte swelling (Figures 1 and 6). Likewise, activation of NF-κB by a mixture of cytokines resulted in a commensurate increase in astrocyte swelling. Further, the observation that BAY 11-7082, an inhibitor of NF-κB, completely diminished the synergistic effect on astrocyte swelling produced by cytokines after prior-sensitization of cultures with ammonia, adds credence to the key role of NF-κB in synergism on cell swelling.

While the time-course of cell swelling and that of NF-κB activation by cytokines were similar, some inconsistencies were noted. Thus, while simultaneous co-treatment of cultures with ammonia and a mixture of cytokines continued to show astrocyte swelling, such treatment unexpectedly resulted in a significant reduction in NF-κB activation as compared to cultures treated with ammonia or with a mixture of cytokines (Figure 5). The reason for this discordance is not known. However, it is possible that the presence of ammonia during the course of cytokine treatment might have interfered with the ability of cytokines to activate NF-κB. It should be noted that this condition is very different from the ammonia pretreatment protocol, as by the time cytokines were added, all of the ammonia had been metabolized and was no longer present in the culture media (data not shown).

We also observed that exposure of cultures to ammonia prior to treatment with a mixture of cytokines caused a marked activation of NF-κB (428%), while the increase in astrocyte swelling was of lesser magnitude (129%). The disparity between these two events is probably due to the fact that the extent of swelling observed (129%) represents the maximal swelling capacity for astrocytes, as we previously documented that maximal increase in cell volume in cultured astrocytes incubated in hypoosmotic media was 120% ([45] and references therein).	

Precisely how NF-κB contributes to astrocyte swelling is not clear. NF-κB is known to activate various genes, including inducible nitric oxide synthase (iNOS) and NADPH oxidase (NOX) [31], whose products nitric oxide, superoxide and peroxynitrite have been shown to cause astrocyte swelling [46-48]. Additionally, NF-κB is known to activate phospholipase A2 (PLA2) as well as cycooxygenase-2 (COX-2) [31], the products of which include arachidonic acid and prostaglandin E2, metabolites capable of inducing astrocyte swelling [49]. Consistent with this view, recent studies have also shown that ammonia activates iNOS, NOX, PLA2 and COX-2 and that inhibition of these enzymes diminished ammonia-induced astrocyte swelling [24,50-52]. Nevertheless, the precise pathway(s) by which NF-κB activation contributes to astrocyte swelling remains to be established.

## Conclusions

TNF-α, IL-1β, IL-6 and IFN-γ, individually and in combination, caused cell swelling in cultured astrocytes and activation of NF-κB. Treatment of cultures with BAY 11-7082 significantly inhibited cytokine-mediated astrocyte swelling indicating a role of NF-κB in such swelling. Additionally, sensitization of cultures with ammonia prior to treatment with cytokines potentiated both cell swelling and NF-κB activation. BAY 11-7082, an inhibitor of NF-κB, completely diminished astrocyte swelling. Altogether, our findings demonstrate a critical role of NF-κB in the potentiation of cell swelling created by ammonia pretreatment followed by exposure to a mixture of cytokines. These findings provide a potential explanation for the exacerbation of brain edema in the setting of sepsis/inflammation, a frequent complication of ALF. Targeting NF-κB may provide a useful strategy for the treatment of the brain edema associated with ALF.

## Competing interests

The authors declare that they have no conflicts of interest either financially or non-financially in this study.

## Authors' contributions

KVR, ARJ, performed major parts of the experiments and XY and VMA assisted in some of the experiments. KVR, ARJ and MDN designed the experiments, analyzed the data and wrote the manuscript. All authors read and approved the final version of the manuscript.
